# Asparagus racemosus Root Extract (SheVari4®) Alleviates Menopausal Symptoms in Pre-, Peri-, and Post-menopausal Healthy Women

**DOI:** 10.7759/cureus.106987

**Published:** 2026-04-13

**Authors:** Ananya Swaroop, Anand Swaroop

**Affiliations:** 1 Biostatistics, Cepham Inc., Somerset, USA; 2 Biochemistry, Cepham Inc., Somerset, USA

**Keywords:** asparagus racemosus, herbal extract, menopause rating scale, menopause symptoms, peri-menopause, post-menopause, pre-menopause, shatavari, shevari4, women’s health

## Abstract

Background

Menopause is a significant transitional phase in women’s lives, characterized by hormonal fluctuations that often entail quality-of-life-altering symptoms. These can include hot flashes, mood changes, and sleep disturbances, among others. Though hormone replacement therapy (HRT) may alleviate some menopausal symptoms, it is not appropriate for all individuals. The Shatavari plant (*Asparagus racemosus*) has long been utilized in traditional Ayurvedic medicine to support women's reproductive health, though rigorous clinical evidence for its efficacy remains limited.

Methods

In this study, we evaluated a standardized *A. racemosus* root extract (SheVari4®) in improving menopausal symptoms across somatic, psychological, and urogenital domains. A total of 60 healthy (pre-, peri-, and post-menopausal) women aged 40-55 years were recruited and randomized into two groups to receive daily doses of either 100 mg of placebo or SheVari4® via double-blinded administration. Menopausal symptoms were assessed at baseline (pre-treatment), and at four and eight weeks (post-treatment), using the Menopause Rating Scale (MRS).

Results

Confirming successful randomization, both groups reported similar baseline MRS scores (~15 on a 44-point scale). However, post-treatment, the SheVari4® group demonstrated a significant reduction in menopausal symptom severity at week 4 (43% decrease) and at week 8 (71% decrease, linear mixed model (LMM) analysis, p < 0.001). Additionally, several parameters across MRS subscales showed significant improvement with SheVari4®, including hot flash occurrence and sleep quality (both >90% improved at week 8). Adverse events were mild, transient, and comparable between groups, with gastrointestinal discomfort being the most common.

Conclusion

These findings suggest that SheVari4® may represent a promising botanical alternative for women seeking non-HRT approaches to manage menopausal symptoms.

## Introduction

Shatavari (*Asparagus racemosus*) is a woody, climbing under-shrub widely cultivated in tropical and subtropical regions of India, Africa, and Australia [1]. Dried succulent tuberous root preparations of Shatavari have a rich history of usage in traditional Indian Ayurvedic medicine to benefit female reproductive health, as an immunomodulatory tonic, and as an adaptogen [2]. These pharmacological properties are attributed to root bioactives, including steroidal saponins (primarily Shatavarin I and IV), alkaloids, isoflavones, flavonoids, sapogenins, and glycosides [3]. Though Shatavari’s beneficial effects as a galactagogue, hepatoprotective agent, anti-tumor promoter, etc., have been well characterized in studies employing animal models, its investigation in human clinical trials is limited [4,5].

One underexplored application of Shatavari is in alleviating menopausal symptoms. In vitro and in silico studies demonstrate binding affinity of Shatavarins with several estrogen receptors (ER α, β, γ), follicle-stimulating hormone (FSH) receptor, luteinizing hormone (LH) receptor, and progesterone [6,7]. Given that the menopausal transition is hallmarked by the decline in ovarian production of estrogen and progesterone [8], external stimulation of these signaling pathways may relieve symptoms associated with hormone fluctuations. In vitro studies show phytoestrogenic compounds Rutin and Shatavarin IV in *A. racemosus* exert anti-proliferative activity by effectively binding to ERα, suggesting potential as an anti-cancer compound [9]. Further, in postmenopausal women with signs of sarcopenia and osteoporosis due to decreased estrogen, Shatavari supplementation improved handgrip strength [10,11] and decreased bone-turnover markers, with concurrent reduced inflammatory and oxidative stress [12]. Though evidence suggests Shatavari exerts its phytoestrogenic effects through both direct modulation of estrogen receptors and indirectly by affecting pathways that influence estrogen availability, exact mechanisms remain to be elucidated [13].

Fueled by a global increase in life expectancy, it is estimated that most women who reach menopause between 49 and 52 years of age will spend ~40% of their lives in postmenopause [14]. Thus, there is a pressing need for the development and research of novel compounds to meet this increasing demand. The pre-, peri-, and post-menopausal space presents a promising opportunity for the introduction of new products. This is due to potential complications associated with existing menopausal therapeutics and increased interest towards complementary and integrative therapeutic solutions for menopausal symptoms [15,16]. Based on Shatavari’s traditional usage and evidence from literature supporting its effects as a phytoestrogen, improver of ovarian and uterine physiology, and stress reducer, we sought to formally investigate its efficacy in a clinical trial. In this study, we examine the ability of SheVari4® (*A. racemosus* root water-ethanol extract containing 5% Shatavarin IV) in improving menopausal symptoms in pre-, peri-, and post-menopausal women over eight weeks, using a self-reported questionnaire assessment (Menopause Rating Scale (MRS) - primary objective) and assaying for safety and tolerability (secondary objective).

## Materials and methods

Study overview and ethics

This was a prospective, single-center, randomized, placebo-controlled, double-blind, parallel-group study conducted at a tertiary care center in New Delhi, India. This study enrolled pre-, peri-, and postmenopausal women as assessed by MRS survey between December 2024 and March 2025. All study procedures were conducted under the oversight of a practicing gynecologist and obstetrician in conjunction with a team of research associates and coordinators. The study design and procedures were reviewed by the institutional ethics committee (approval IEC/SNH/2025/01/#11035), and this clinical trial was registered with the Clinical Trial Registry of India (CTRI/2025/02/#079989). The study adhered to ethical principles outlined in the Declaration of Helsinki and was conducted in a manner consistent with good clinical practices [17].

Freely given informed consent was obtained from all subjects prior to participation, and all participants had the right to leave the study at any time or request their data be rescinded without penalty. Participants were informed about any new information that emerged during research that may change their assessment of the risk/benefit of participating. The welfare of all participants was monitored throughout the course of the study for adverse reactions and changes in clinical status, ensuring treatment and/or study removal if applicable. Subject privacy and confidentiality were maintained by de-identifying, restricting access, and ensuring proper recording, handling, and storage of all information.

Study population

The study enrolled 60 healthy women between 40 and 55 years of age, distributed across premenopausal, perimenopausal, and postmenopausal groups. Participants underwent complete blood count (CBC) testing at baseline and at week 8 post-treatment to assess overall health (Table 1). A total sample size of 60 subjects (30 per treatment group) was calculated using 80% power (β = 0.2), 95% confidence interval (α = 0.05), effect size E = 0.7, with equal proportions of subjects in each group, considering a projected dropout/exclusion rate of ~20%. Inclusion criteria utilized in recruitment entailed a body mass index of 18.5-29.9 (calculated as weight (kg) divided by height (m)²), resting systolic blood pressure <140 mmHg, diastolic blood pressure <90 mmHg, resting heart rate <90 beats per minute, and amenorrhea for the last 12 consecutive months for postmenopausal women. Participants were identified as pre-, peri-, or postmenopausal by a gynecologist/obstetrician based on self-reported clinical history and symptoms. Criteria used to evaluate women included age, date of last menstrual cycle, cycle regularity, symptoms, and past hormonal testing if previously performed. Exclusion criteria employed in this study included current bladder dysfunction or urinary tract issues, history of breast, uterine, ovarian, or cervical cancers, use of hormone replacement therapy (HRT) or oral contraceptives, history of hysterectomy, regular consumption of soy-containing foods, and current smoking or excessive alcohol consumption.

**Table 1:** Summary data and results of independent samples t-test for complete blood test results by treatment group. Independent samples t-test was conducted with two-sided p-values reported.

Parameter	Placebo Baseline, Mean ± SEM	Placebo Week 8, Mean ± SEM	SheVari4^®^ Baseline, Mean ± SEM	SheVari4^®^ Week 8, Mean ± SEM	Test Statistic and p-value Between Groups, Baseline	Test Statistic and p-value Between Groups, Week 8
Red Blood Cell Count (RBC ×10⁶/μL)	4.18 ± 0.05	4.17 ± 0.05	4.16 ± 0.04	4.13 ± 0.04	t_(58) _= 0.199, p = 0.843	t_(58) _= 0.517, p = 0.607
Hemoglobin (g/dL)	12.71 ± 0.17	12.64 ± 0.16	12.70 ± 0.14	12.68 ± 0.15	t_(58) _= 0.075, p = 0.940	t_(58) _= -0.167, p = 0.868
Hematocrit %	38.25 ± 0.50	38.36 ± 0.50	38.07 ± 0.43	38.02 ± 0.44	t_(58) _= 0.282, p = 0.779	t_(58) _= 0.510, p = 0.612
Mean Corpuscular Volume (fL)	91.46 ± 0.52	91.01 ± 0.78	91.05 ± 0.52	90.67 ± 0.73	t_(58) _= 0.551, p = 0.584	t_(58) _= 0.311, p = 0.757
Mean Corpuscular Hemoglobin (pg)	30.39 ± 0.19	30.14 ± 0.24	30.59 ± 0.18	30.58 ± 0.25	t_(58) _= -0.755, p = 0.453	t_(58) _= -1.279, p = 0.206
Mean Corpuscular Hemoglobin Concentration (g/dL)	33.34 ± 0.12	33.39 ± 0.20	33.41 ± 0.10	33.53 ± 0.16	t_(58) _= -0.482, p = 0.631	t_(58) _= -0.535, p = 0.595
Platelets (×10³/μL)	261.23 ± 8.76	260.53 ± 8.62	256.67 ± 7.93	256.90 ± 7.76	t_(58) _= 0.386, p = 0.701	t_(58) _= 0.313, p = 0.755
White Blood Count (×10³/μL)	6.72 ± 0.23	6.73 ± 0.24	6.50 ± 0.19	6.44 ± 0.19	t_(58) _= 0.740, p = 0.462	t_(58) _= 0.939, p = 0.352
Neutrophils (%)	58.37 ± 0.58	58.20 ± 0.58	58.93 ± 0.64	58.84 ± 0.69	t_(58) _= -0.656, p = 0.514	t_(58) _= -0.706, p = 0.483
Lymphocytes (%)	32.31 ± 0.57	32.21 ± 0.55	31.54 ± 0.69	31.63 ± 0.73	t_(58) _= 0.863, p = 0.391	t_(58) _= 0.631, p = 0.531
Monocytes (%)	5.97 ± 0.21	5.97 ± 0.22	6.24 ± 0.14	6.24 ± 0.15	t_(58) _= -1.062, p = 0.292	t_(58) _= -1.043, p = 0.301
Eosinophils (%)	2.77 ± 0.15	2.78 ± 0.16	2.76 ± 0.14	2.75 ± 0.14	t_(58) _= 0.080, p = 0.937	t_(58) _= 0.109, p = 0.913
Basophils (%)	0.59 ± 0.03	0.59 ± 0.03	0.52 ± 0.04	0.52 ± 0.04	t_(58) _= 1.343, p = 0.184	t_(58) _= 1.343, p = 0.184
Absolute Neutrophil Count (×10³/μL)	3.93 ± 0.15	3.94 ± 0.15	3.83 ± 0.11	3.82 ± 0.12	t_(58) _= 0.558, p = 0.579	t_(58) _= 0.662, p = 0.510
Absolute Lymphocyte Count (×10³/μL)	2.17 ± 0.08	2.17 ± 0.08	2.06 ± 0.08	2.07 ± 0.08	t_(58) _= 0.989, p = 0.327	t_(58) _= 0.922, p = 0.360
Reticulocytes (%)	1.41 ± 0.05	1.41 ± 0.05	1.33 ± 0.06	1.32 ± 0.06	t_(58) _= 0.948, p = 0.347	t_(58) _= 1.079, p = 0.285
Ferritin (ng/mL)	42.01 ± 3.78	41.97 ± 3.73	45.39 ± 3.16	45.66 ± 3.12	t_(58) _= -0.685, p = 0.496	t_(58) _= -0.758, p = 0.452
Erythrocyte Sedimentation Rate (mm/hr)	13.60 ± 1.19	13.67 ± 1.21	15.33 ± 0.91	15.37 ± 0.93	t_(58) _= -1.157, p = 0.252	t_(58) _= -1.110, p = 0.272

Study intervention

The test substance, SheVari4®, is a proprietary ethanolic extract of *A. racemosus* (Shatavari) root, standardized to contain not less than 5% Shatavarin IV, a steroidal saponin with the molecular formula C₄₅H₇₄O₁₇. Third-party analytical verification by Alkemist Labs (Garden Grove, CA, USA), conducted via validated liquid chromatography-mass spectrometry (LCMS) methodology (Method ATM-815-0338), has consistently confirmed Shatavarin IV content across multiple production batches ranging from 6.687% to 7.094%, well exceeding the established specification. SheVari4® is enrolled in the Alkemist Assured™ program, which requires ongoing identity, potency, and purity confirmation testing of raw materials and blended ingredients, providing an additional layer of quality assurance. The clinical significance of Shatavarin IV has been substantiated in a peer-reviewed study published in the Journal of the American Nutrition Association [18], which demonstrated that SheVari4® exerts potent anti-inflammatory and antioxidant effects in LPS-treated SH-SY5Y neuroblastoma cells via the TrkB-BDNF signaling axis, establishing a mechanistic basis for its neuroprotective potential. Participants in this study were randomized in a 1:1 ratio into the following groups: (A) SheVari4® group, administered one capsule of SheVari4® (100 mg) daily, or (B) Placebo group, administered one capsule of placebo (100 mg brown rice starch) daily. Treatment was continued for eight weeks, with participants instructed to take a capsule during their evening meal with one cup of water.

Randomization and blinding

The completed study population of 60 subjects was assigned in Excel (Microsoft® Corp., Redmond, WA, USA) following a one-to-one allocation, with 30 subjects in treatment group 1 and 30 in treatment group 2, fully satisfying the permuted block randomization plan. The design specified a block size of four across 15 sequential blocks, targeting 30 subjects per arm at completion. The outcome of 30 completers in each arm corresponds precisely to 15 intact blocks with no remainder, confirming that the allocation sequence was executed without structural imbalance or allocation bias. The deliberate enrollment of 64 subjects in excess of the 60-subject minimum served as a pre-specified buffer against anticipated attrition, and the differential dropout of one subject from treatment group 1 and 3 from treatment group 2 resolved the over-enrollment at study close, restoring exact allocation balance. The randomization was therefore validated as satisfactorily meeting all requirements of the permuted block design. Investigators were blinded to subject identity by replacing identifying data with unique IDs via lookup tables and using data masking in Excel prior to block randomization. Investigators remained blinded throughout the study procedure, as to the identity of the treatment groups, and were only unblinded following completion of statistical analysis.

Outcome measures

The primary endpoints in this study utilized the MRS administered at baseline and at four and eight weeks post-intervention. The MRS is a globally utilized and validated questionnaire designed to assess the severity of both physical and psychological symptoms associated with menopause [19]. Formal permission and licensing for commercial clinical research were obtained and paid for by the study sponsor from Zentrum für Epidemiologie und Gesundheitsforschung Berlin GmbH (ZEG Berlin GmbH), the home institution of the copyright holder of the MRS, Dr. Lothar A.J. Heinemann. The MRS comprises 11 parameters covering three subscales: somatic (questions 1, 2, 3, and 11), psychological (questions 4-7), and urogenital (questions 8-10). Symptoms assessed include hot flashes/sweating, cardiac issues, sleep problems, depressive mood, irritability, anxiety, exhaustion, sexual problems, bladder issues, vaginal dryness, and joint and muscular pain. Each symptom is self-assessed by subjects and ranked on a scale of 0 to 4, with 0 indicating the absence of the symptom and 4 representing extremely severe symptoms. Hence, the total possible score for the entire questionnaire is 44, with overall symptom severity scored thusly: 0-4 (none), 5-8 (mild), 9-15 (moderate), 16+ (severe). Secondary endpoints for this study evaluated changes in weight and blood pressure, if any, and assessed the safety and tolerability of SheVari4®, recording incidence and severity of any adverse events.

Statistical analysis

Data analysis was conducted using IBM SPSS Statistics for Windows, Version 30.0 (Released 2024; IBM Corp., Armonk, NY, USA). Quantitative parameters were described using frequencies and percentages. No replacement of missing values was performed, nor were any subjects/data points removed from analysis. Outcome variables were assessed for normality, following which, parametric tests were performed for normally distributed data, and non-parametric tests were performed for data that were non-normal after transformation. Comparative analyses were conducted between treatment groups for all endpoints. Independent student’s t-tests were used to compare individual outcome variables between treatment groups at a single time point. Linear mixed model (LMM) analysis with age as a covariate was used to study the effects of time (baseline, week 4, and week 8), and treatment (SheVari4® or placebo) on outcome variables to account for repeated measures. Statistical significance was set a priori at α = 0.05.

## Results

**Figure 1:**
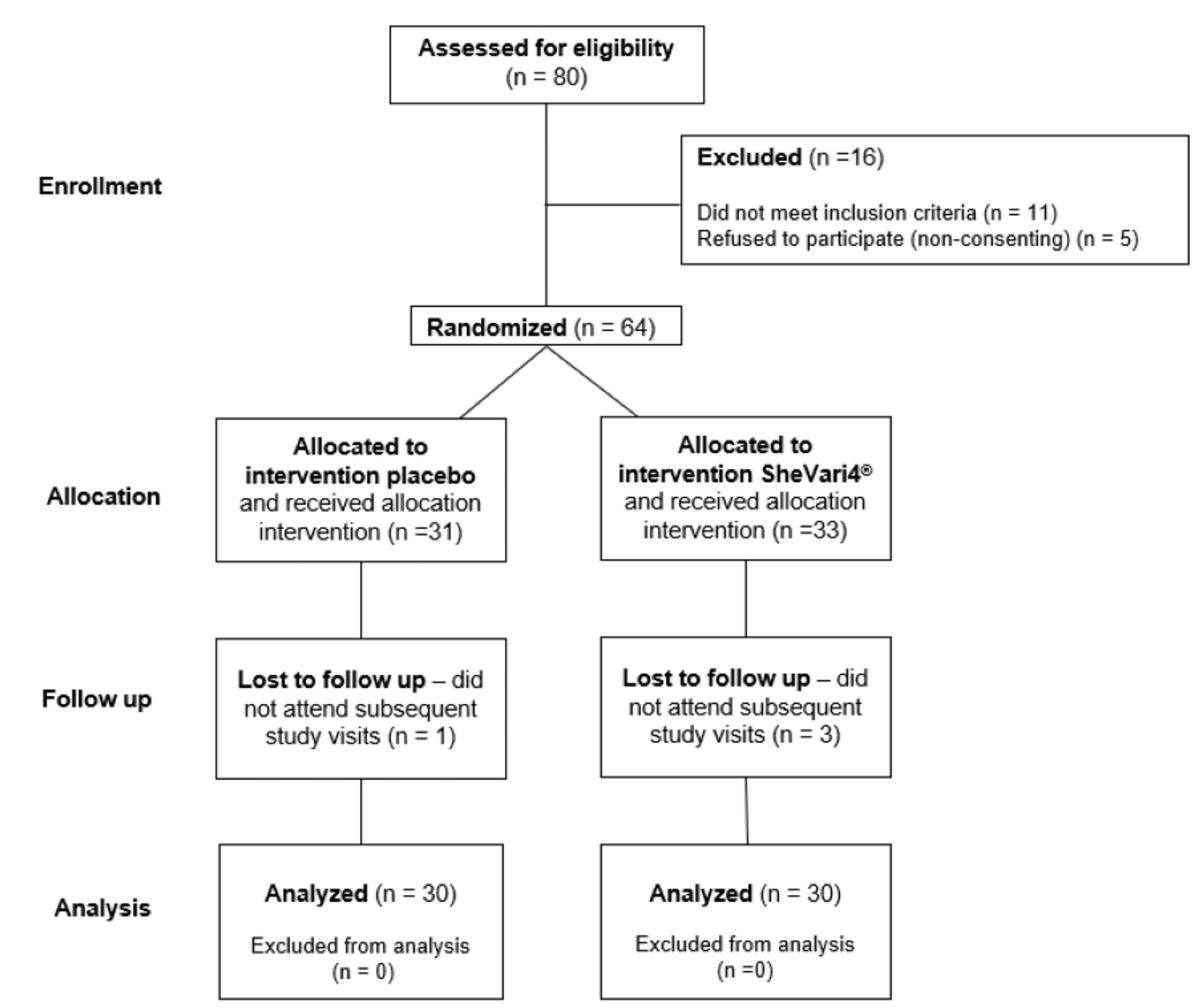
CONSORT flow diagram.

**Figure 2:**
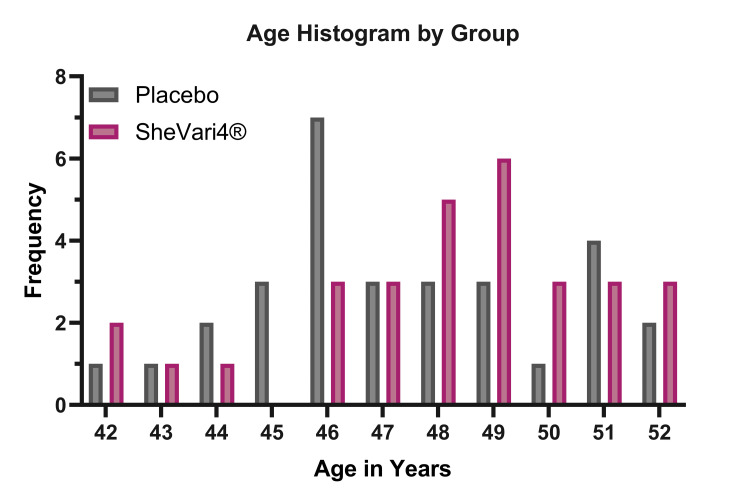
Histogram of ages by treatment group shows roughly equal distribution.

A total of 80 women were screened for eligibility, of whom 64 met the inclusion criteria and were subsequently randomized to receive either SheVari4® or placebo (Figure 1). The completion rate was 93.3%, with two participants from the SheVari4® group and two from the placebo group withdrawing from the study, yielding a total of 60 participants (30/group). The mean age of participants was 48.6 ± 4.3 years in the SheVari4® group and 49.1 ± 4.7 years in the placebo group (Figure 2). Both groups were well-matched for demographic and clinical characteristics at baseline.

**Figure 3:**
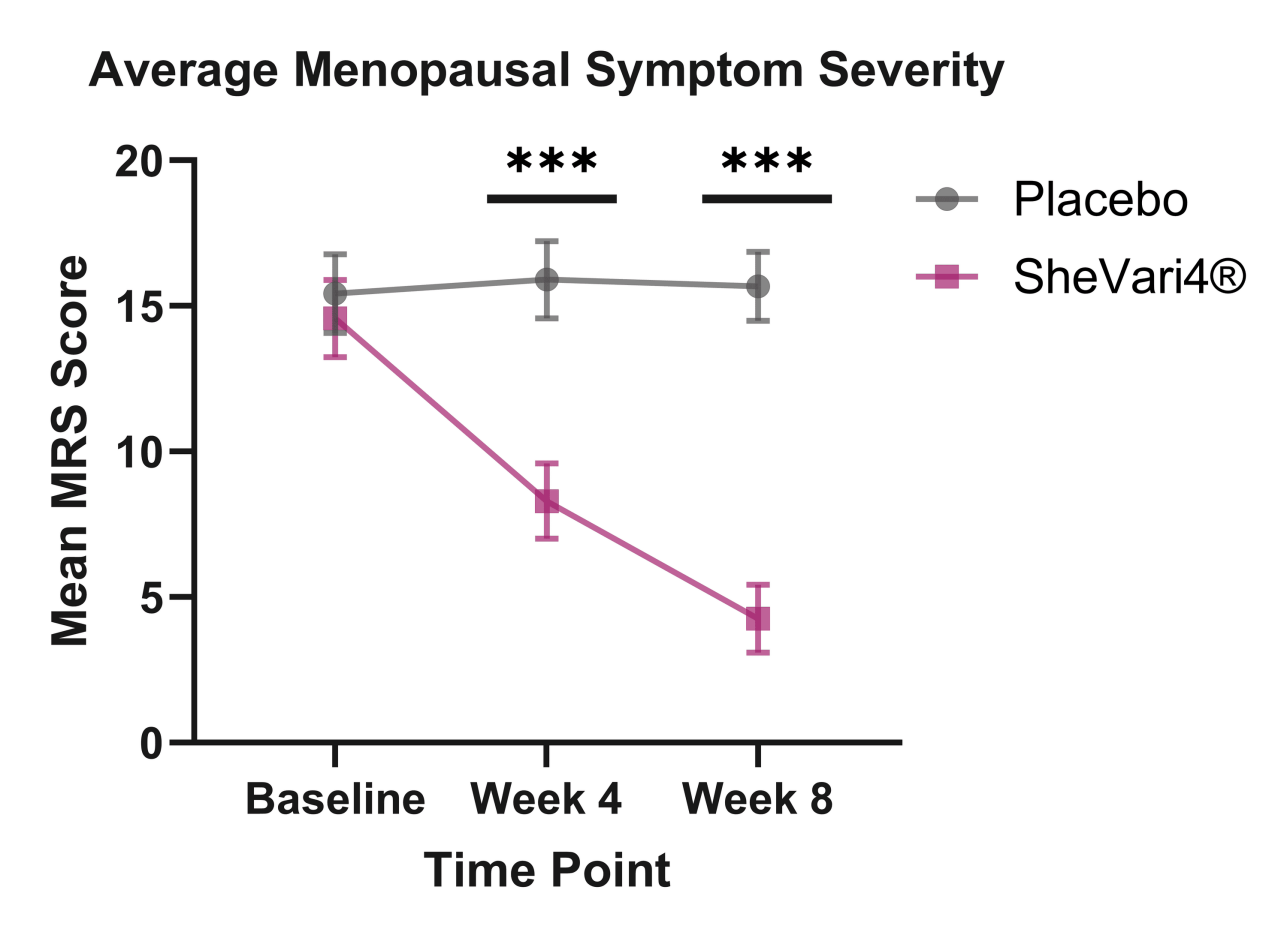
SheVari4® administration reduces menopausal symptoms at four- and eight-weeks post-administration. *** denotes p < 0.001. SheVari4® supplementation significantly reduces menopausal symptoms in comparison to placebo at both four and eight weeks following daily consumption. Mean ± standard error of the mean (SEM) graphed. Using the following independent variables: time point (baseline, week 4, and week 8), treatment group (SheVari4®, placebo), and age, linear mixed model (LMM) analysis was conducted, yielding a significant interaction of Time Point × Group F_(2,63.53)_ = 23.98, p < 0.001.

**Figure 4:**
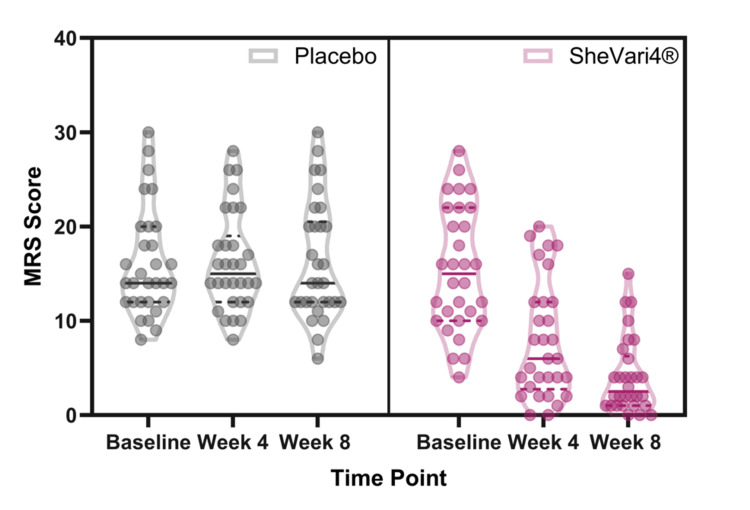
Violin plots showing the spread of total MRS scores across groups and decrease in menopausal symptoms for SheVari4® group. MRS: Menopause Rating Scale

**Figure 5:**
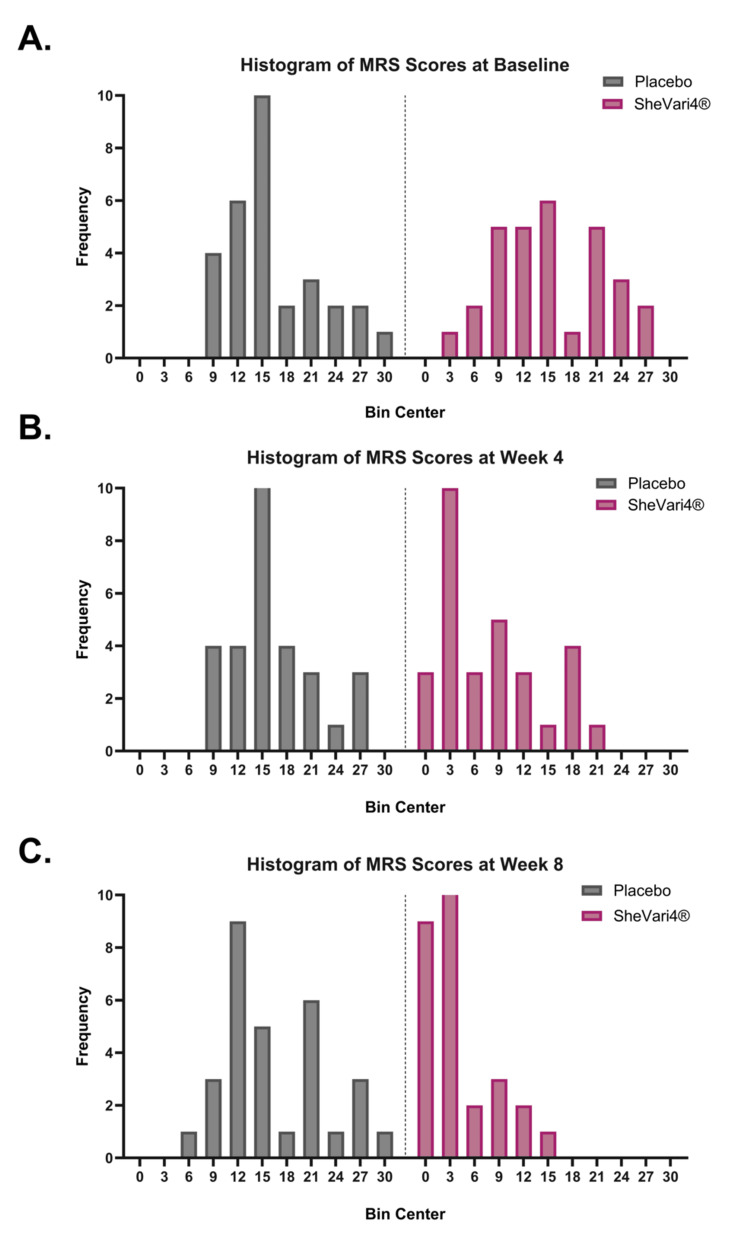
Histograms of MRS scores by treatment group at each time point. MRS scores at (A) baseline, (B) week 4, and (C) week 8 demonstrate a gradual shift towards less severe menopausal symptom severity, i.e., lower scores, in the SheVari4® group compared to placebo. Scores are binned in intervals of 3. MRS: Menopause Rating Scale

Through the duration of the study, all participants were assessed for physical and psychological menopausal symptoms using the clinically validated MRS [19] at baseline (day 0), week 4 (day 28), and at week 8 (day 56). Participants assigned to the SheVari4® group showed statistically significant improvements in total MRS scores compared to the placebo group at both four and eight weeks following daily consumption (Figure 3 and Table 2). This trend favoring SheVari4® administration is also easily visualized using violin plots (Figure 4) and frequency histograms of MRS score by time point across groups (Figure 5).

**Table 2:** Means and significance values of pairwise comparisons following analysis on the effect of supplement administration on Menopause Rating Scale (MRS) scores. Statistical analysis utilized linear mixed model (LMM), revealing a significant Time Point × Group interaction; F_(2,63.53)_ = 23.98, p < 0.001.

Time Point	Placebo Group (Mean ± SEM)	SheVari4^®^ Group (Mean ± SEM)	Pairwise Comparisons p-values
Baseline	15.42 ± 1.35	14.57 ± 1.32	0.655
Week 4	15.92 ± 1.32	8.29 ± 1.29	<0.001
Week 8	15.68 ± 1.19	4.26 ± 1.16	<0.001

To analyze whether SheVari4® had differential effects based on menopausal symptom severity at baseline, we split MRS scores post-treatment into two groups: women reporting relatively low (0-15) vs high (16+) scores at baseline (Table 3). This split was performed based on visual binning (SPSS) based on MRS score values. Percent decrease scores compared to baseline were calculated for four- and eight-week time points and revealed that SheVari4® supplementation had similar effects on women reporting either mild-moderate or severe menopausal symptoms. In contrast, placebo administration had very little effect on MRS score (<5%) in both groups of women.

**Table 3:** Analysis of MRS sub-scores shows several parameters are significantly improved with SheVari4®. MRS sub-score LMM analysis, with corresponding pairwise comparisons (parameters that significantly vary between placebo and SheVari4® are bolded). MRS: Menopause Rating Scale; LMM: Linear Mixed Model

Sub-score	Group x Time Point Two-Way F-score	F-score p-value	Time Point	Placebo Group (Mean ± SEM)	SheVari4® Group (Mean ± SEM)	Pairwise Comparisons p-value
Hot Flash Severity	F_(2,39)_ = 61.74	p < 0.001	Baseline	2.35 ± 0.21	2.57 ± 0.21	p = 0.46
			Week 4	2.48 ± 0.23	1.09 ± 0.23	p < 0.001
			Week 8	2.27 ± 0.17	0.22 ± 0.17	p < 0.001
Cardiac Discomfort	F_(2,39)_ = 1.54	p = 0.23	Baseline	0.17 ± 0.11	0.22 ± 0.11	p = 0.80
			Week 4	0.06 ± 0.05	0.00 ± 0.05	p = 0.45
			Week 8	0.17 ± 0.09	0.02 ± 0.08	p = 0.30
Sleep Problems	F_(2,39)_ = 12.25	p < 0.001	Baseline	2.80 ± 0.24	2.25 ± 0.24	p = 0.12
			Week 4	2.86 ± 0.28	0.68 ± 0.27	p < 0.001
			Week 8	2.48 ± 0.23	0.14 ± 0.23	p < 0.001
Depressive Mood	F_(2,39)_ = 2.79	p = 0.07	Baseline	0.96 ± 0.25	0.62 ± 0.25	p = 0.35
			Week 4	1.11 ± 0.24	0.22 ± 0.23	p = 0.01
			Week 8	1.10 ± 0.19	0.06 ± 0.19	p < 0.001
Irritability	F_(2,39)_ = 3.12	p = 0.06	Baseline	0.86 ± 0.23	0.76 ± 0.22	p = 0.75
			Week 4	1.05 ± 0.20	0.34 ± 0.19	p = 0.013
			Week 8	0.92 ± 0.19	0.13 ± 0.19	p = 0.006
Anxiety	F_(2,39)_ = 8.22	p = 0.001	Baseline	1.34 ± 0.23	1.26 ± 0.23	p = 0.81
			Week 4	1.34 ± 0.24	0.58 ± 0.24	p = 0.033
			Week 8	1.47 ± 0.20	0.19 ± 0.19	p < 0.001
Physical, Mental Exhaustion	F_(2,39)_ = 5.838	p = 0.006	Baseline	2.75 ± 0.24	2.53 ± 0.24	p = 0.53
			Week 4	2.69 ± 0.33	1.93 ± 0.32	p = 0.11
			Week 8	2.87 ± 0.28	1.48 ± 0.27	p < 0.001
Sexual Problems	F_(2,39)_ = 10.203	p < 0.001	Baseline	1.29 ± 0.30	1.56 ± 0.29	p = 0.52
			Week 4	1.35 ± 0.29	1.20 ± 0.28	p = 0.71
			Week 8	1.67 ± 0.26	0.60 ± 0.25	p = 0.005
Bladder Problems	F_(2,39)_ = 0.589	p = 0.560	Baseline	0.20 ± 0.12	0.10 ± 0.12	p = 0.58
			Week 4	0.23 ± 0.10	0.00 ± 0.10	p = 0.12
			Week 8	0.20 ± 0.10	0.00 ± 0.10	p = 0.19
Vaginal Dryness	F_(2,39)_ = 5.238	p = 0.10	Baseline	2.00 ± 0.25	2.17 ± 0.25	p = 0.65
			Week 4	2.03 ± 0.28	1.84 ± 0.27	p = 0.63
			Week 8	2.00 ± 0.23	1.17 ± 0.23	p = 0.014
Joint, Muscular Pain	F_(2,39)_ = 0.949	p = 0.396	Baseline	0.72 ± 0.19	0.53 ± 0.18	p = 0.49
			Week 4	0.72 ± 0.18	0.42 ± 0.17	p = 0.23
			Week 8	0.60 ± 0.16	0.25 ± 0.15	p = 0.12

Next, to further understand which individual questions on the MRS survey were driving reduced scores in the SheVari4® group, we conducted a similar LMM analysis on individual questions that were ranked by participants on a scale of 0-4, encompassing mild, moderate, severe, or extremely severe categories (Table 4). Among these, hot flash severity, sleep problems, depressive mood, irritability, and anxiety showed improvement as early as four weeks, with physical and mental exhaustion, sexual problems, and vaginal dryness improving at eight weeks with SheVari4® supplementation. This trend was reflected in the percentage change from baseline, where several menopausal symptoms showed >40% change towards less severity (Table 5).

**Table 4:** Mean percent decrease for significantly different MRS sub-score parameters. Cells with at least 40% change are bolded. MRS: Menopause Rating Scale

Sub-score	Placebo % Change From Baseline	SheVari4® % Change From Baseline
Hot Flash Severity	Week 4 - 5.53% increase	Week 4 - 57.59% decrease
	Week 8 - 3.40% decrease	Week 8 - 91.44% decrease
Sleep Problems	Week 4 - 2.14% increase	Week 4 - 69.78% decrease
	Week 8 - 11.43% decrease	Week 8 - 93.38% decrease
Anxiety	Week 4 - 0% change	Week 4 - 53.97% decrease
	Week 8 - 9.70% increase	Week 8 - 84.92% decrease
Physical, Mental Exhaustion	Week 4 - 2.18% decrease	Week 4 - 23.72% decrease
	Week 8 - 4.36% increase	Week 8 - 41.50% decrease
Sexual Problems	Week 4 - 4.65% increase	Week 4 - 23.08% decrease
	Week 8 - 29.46% increase	Week 8 - 61.54% decrease
Vaginal Dryness	Week 4 - 1.5% increase	Week 4 - 15.21% decrease
	Week 8 - 0% change	Week 8 - 46.08% decrease

**Table 5:** MRS scores improve in proportion to time administered with SheVari4® treatment regardless of baseline score. MRS: Menopause Rating Scale

Condition	Baseline (Mean ± SEM)	Week 4 (Mean ± SEM)	% Change From Baseline	Week 8	% Change From Baseline
Placebo MRS 0-15 Baseline Scores	12.18 ± 0.49	12.76 ± 0.58	4.76%	11.88 ± 0.64	2.46%
SheVari4® MRS 0-15 Baseline Scores	9.93 ± 0.75	4.60 ± 0.97	53.68%	2.53 ± 0.56	74.52%
Placebo MRS 16+ Baseline Scores	21.23 ± 1.31	20.85 ± 1.21	1.79%	22.15 ± 1.27	4.33%
SheVari4® MRS 16+ Baseline Scores	20.93 ± 1.01	11.20 ± 1.68	46.49%	5.67 ± 1.23	72.91%

Adverse events reported by participants were mild and transient, resolving without intervention (Table 6). No serious adverse events were reported in either group. We additionally assayed for changes in weight between treatment groups and found no statistically significant differences from baseline in either arm (Table 7). While this short-term safety profile is encouraging, future studies should include longer duration monitoring for late-onset adverse effects and comprehensive laboratory assessments for adverse subclinical effects. Additionally, evaluation of potential herb-drug interactions, especially with commonly used medications in this population, and specific safety assessments in special populations (e.g., women with pre-existing conditions), would benefit the investigation of this therapeutic.

**Table 6:** Summary of adverse events reported by participants.

Adverse Event	SheVari4^®^ Group (n = 30)	Placebo Group (n = 30)
Mild gastrointestinal discomfort	3 (10%)	2 (6.7%)
Headache	2 (6.7%)	3 (10%)
Nausea	1 (3.3%)	2 (6.7%)
Dizziness	0 (0%)	1 (3.3%)

**Table 7:** Summary of independent samples t-test results from analysis of change in weight by treatment group. Independent samples t-test was performed on the difference in values from week 8 to baseline, yielding no significant differences between groups, t(58) = 1.289, p = 0.203.

Group	Baseline (Mean ± SEM)	Week 8 (Mean ± SEM)	Average Difference (Mean ± SEM)
Placebo	64.1 ± 1.6 kg	64.3 ± 1.6 kg	0.2 ± 0.5 kg
SheVari4^®^	63.4 ± 1.5 kg	62.8 ± 1.4 kg	-0.6 ± 0.4 kg

## Discussion

This clinical trial demonstrated that SheVari4® supplementation for both four- and eight-week periods significantly improved menopausal symptoms as measured by the MRS compared to placebo. Additionally, participants taking SheVari4® reported further improvement at the eight-week time point compared to the four-week time point, as indicated by a greater decrease in menopause severity scores. Participants also reported significant improvements in all three MRS subscales (somatic, psychological, and urogenital) in the SheVari4® group. Our findings align with previous literature demonstrating improved Menopause-Specific Quality of Life (MENQOL) scores in healthy older women with use of an Ayurvedic formulation containing 100 mg *A. racemosus* root extract [20], and improved Utian Quality of Life (UQoL) scores in pre- and post-menopausal women given 250 mg of a Shatavari root extract standardized to 5% total Shatavarins [21].

In addition to grossly improved menopausal symptoms when tested at four weeks, we found that improvement in MRS score in women taking SheVari4® was global, irrespective of whether participants reported mild, moderate, or severe menopausal symptoms at baseline. Though further studies using a more comprehensive average baseline score and including more participants with heterogeneous scores need to be conducted, preliminary results suggest SheVari4® exerts diverse effects across psychological, somatic, and urogenital domains, imparting a positive effect on various menopausal symptoms irrespective of severity.

Improvements in psychological symptoms with SheVari4® are particularly noteworthy in this trial, with women reporting reduced depressive mood, irritability, and anxiety as soon as four weeks, and decreased mental and physical exhaustion within eight weeks. Previously published preclinical findings in rodents demonstrated anti-depressive qualities of methanolic *A. racemosus* root extract, evidenced by improved outcomes in forced swim and learned helplessness tests [22]. Mechanistic investigation shows Shatavari root extract can regulate stress response by activating monoaminergic neurotransmission and modulating the hypothalamic-pituitary-adrenal (HPA) axis, decreasing plasma norepinephrine and corticosterone in rodent models [23]. Though detailed examination of Shatavari’s neuropharmacological effects in human studies remains to be conducted, evidence from this study and other clinical trials suggests promising neuromodulatory and adaptogenic benefits of Shatavari supplementation [24,25].

In this study, we noted no improvements in symptoms of cardiac discomfort (unusual awareness of heartbeat, heart skipping, heart racing, and tightness), bladder problems (difficulty in urinating, increased need to urinate, and bladder incontinence), nor in joint and muscular problems (pain in the joints and rheumatoid complaints) when comparing SheVari4® and placebo groups. We believe this is largely attributable to the baseline attributes of the study population, who largely reported little/no or mild cardiac discomfort, bladder issues, and joint/muscular pain. Thus, since symptoms were modest at the outset, there was little margin for improvement available to effectively determine the supplement’s capacity in symptom amelioration. Future studies should thus employ greater numbers of participants with significant reports of cardiac, bladder, and joint/muscular symptoms to conclusively assess SheVari4®’s use in these areas.

We find the safety profile of SheVari4® to be acceptable, with only minor, transient adverse events reported at rates akin to placebo. Together, these findings support the long history of traditional safe use of Shatavari in Ayurvedic medicine for female reproductive health and hormonal balance. This study has several limitations that should be considered when interpreting the results. These include the relatively small sample size population of 60 participants, the two-month duration of the study, the single-center design with limited demographic diversity, and the lack of stratification by menopausal status (i.e., pre-, peri-, or post-) in the analysis. Additionally, studies of nutraceutical ingredients can benefit from the addition of a positive control arm (in addition to a placebo) via HRT treatment or an alternative compound to allow for more comprehensive comparisons. The primary objective of this study was to generate preliminary evidence on the efficacy and safety of SheVari4®, and although our findings are encouraging, future studies should recruit greater numbers and diversity of demographic pools, employ additional study arms, and assess quantitative blood biomarkers to definitively assess SheVari4® in reducing menopausal symptoms. These results can also be complemented by studying women with diverse menopausal presentations and by studying the mechanistic bases (whether via phytoestrogenic or other pathways) underlying Shatavari action.

## Conclusions

In conclusion, SheVari4® supplementation (*A. racemosus* root extract standardized to Shatavarin IV, taken at 100 mg daily for eight weeks) significantly improved menopausal symptoms across multiple domains of the MRS compared to placebo in pre-, peri-, and post-menopausal groups of women. SheVari4® was well-tolerated, with a safety profile comparable to placebo. In reviews of similar studies involving Shatavari root extract, our findings align with emerging evidence, strengthening the therapeutic potential of SheVari4®. Future research needs to be conducted to address the limitations outlined in this report and to better understand the effect of *A. racemosus* on human physiology.
